# Supplementary material for: Export competitiveness network of Chinese provincial agricultural products: E volution, performance, and influencing factors
Source: PLoS One. 2025 Jan 16;20(1):e0316088. doi: 10.1371/journal.pone.0316088 (PMC11737858; doi:10.1371/journal.pone.0316088)
Supplement: Attachment — Submitted filename: Review report PONE-D-24-04543_R2.pdf [file pone.0316088.s007.pdf]

Review report:

I have read the manuscript entitled " Export competitiveness network of Chinese provincial agricultural products: Evolution, performance, and influencing factors." The manuscript can be considered for publication after the following questions and recommendations:

1- The researchers did not indicate what is new and distinctive in the abstract of this manuscript (compared to other previous studies).
2- The authors did not specify the method used to determine the sample size.
3- The idea of the manuscript is based on the exported products to determine which products have the most significant impact on the global market. Therefore, the Pareto chart will help draw a clearer picture for the reader and summarize the manuscript in one chart. The Pareto chart aims to highlight the most important products among a (usually large) group of products.
4- The researchers did not mention why this regression method was used to analyze the results.
