# Supplementary material for: Export competitiveness network of Chinese provincial agricultural products: E volution, performance, and influencing factors
Source: PLoS One. 2025 Jan 16;20(1):e0316088. doi: 10.1371/journal.pone.0316088 (PMC11737858; doi:10.1371/journal.pone.0316088)
Supplement: Attachment — Submitted filename: Response to Reviewers.docx [file pone.0316088.s003.docx]

We would like to express our gratitude to the editor for their valuable suggestions, which have contributed to the enhancement of the manuscript. Additionally, we extend our thanks to the editors for affording us another opportunity.

The email dated February 5, 2024, highlights two issues requiring attention.

1, Uploading a Track Changes version of the manuscript file, which highlights any changes made to the previous submission (PONE-D-24-00654).

All modifications made in the updated submission (PONE-S-24-05885) are reflected in the previous submission (PONE-D-24-00654). Please refer to the document "manuscript-Track Changes version.docx" for details.

The modifications include adjustments to the structural framework, format, and details in the manuscript in accordance with the submission guidelines (website: <http://journals.plos.org/plosone/s/submission-guidelines>). The manuscript has undergone extensive revisions informed by two documents: "Download sample title, author list, and affiliations page (PDF)" and "Download sample manuscript body (PDF)".

We found the “scientific editing service” on the web and edited the English. The English writing has been revised using Youdao's English correction writing function, proofread using Wordvice AI, and the English writing and grammatical expression have been modified.

2, Please remove your figures from within your manuscript file, leaving only the individual TIFF/EPS image files.

The figures have been removed from the manuscript file and have been uploaded in TIFF/EPS format.
