# Supplementary material for: Export competitiveness network of Chinese provincial agricultural products: E volution, performance, and influencing factors
Source: PLoS One. 2025 Jan 16;20(1):e0316088. doi: 10.1371/journal.pone.0316088 (PMC11737858; doi:10.1371/journal.pone.0316088)
Supplement: Attachment — Submitted filename: Reviewer_Comments_PONE-D-24-04543.docx [file pone.0316088.s004.docx]

**Reviewer's Comment:**

The manuscript, titled "Research on the evolution of the structure and influencing factors of the export competitiveness network of Chinese provincial agricultural products – Based on a two-mode network perspective," has examined the changes in agricultural exports and the factors that impact their export competitiveness.

This is an excellent paper, and it deals with an important and timely issue using quantitative analytical tools. They have rightly used network analysis. However, the authors must address the following issues to enhance the paper's quality, readability, and communication.

- *Title of the paper*: The title of the manuscript is very long. The title of an article is very important to enhance its searchability, reach the audience, engage readers, influence their thoughts, and increase the probability of citations. The title is expected to be understandable to people, even those who are not experts in the field. Therefore, to reflect the content and make it easily understandable, the title may be revised as "**Export competitiveness network of Chinese provincial agricultural products: Evolution, performance, and influencing factors**."
- *Strengthen the discussion section*: The authors cited other studies, mainly in the introduction and methodology section, to highlight the importance of examining an issue. They have not compared the results of the present study with other studies dealing with similar topics, such as the export competitiveness of agricultural products (for example, Long, 2020) and the influence of Belt and Road on competitive advantage (for example, Feng et al., 2020). They can also cite the results of studies on trade competitiveness and international sister cities. For example, the manuscript has cited the study by Ramasamy and Cremer (1998), who analyzed the economic role of international sister-city relationships between New Zealand and Asia. Readers and policymakers will appreciate it if the authors cite the results of such studies. Thoughtful comparison always enhances the quality and impact of our research.

**References**

Feng, L., Xu, H., Wu, G., Zhao, Y., & Xu, J. (2020). Exploring the structure and influence factors of trade competitive advantage network along the Belt and Road. *Physica A: Statistical Mechanics and Its Applications*, *559*, 125057.

Long, Y. (2021). Export competitiveness of agricultural products and agricultural sustainability in China. *Regional Sustainability*, *2*(3), 203-210.

Ramasamy, B., & Cremer, R. D. (1998). Cities, commerce and culture: The economic role of international sister‐city relationships between New Zealand and Asia. *Journal of the Asia Pacific Economy*, *3*(3), 446-461.
