# Supplementary material for: Export competitiveness network of Chinese provincial agricultural products: E volution, performance, and influencing factors
Source: PLoS One. 2025 Jan 16;20(1):e0316088. doi: 10.1371/journal.pone.0316088 (PMC11737858; doi:10.1371/journal.pone.0316088)
Supplement: Attachment — Submitted filename: Response to Reviewers.docx [file pone.0316088.s005.docx]

We wish to thank the reviewer 1 for her/his detailed comments and suggestions that helped to tremendously improve the manuscript.

Reviewer #1: Greetings! This manuscript reveals the foreign trade network of agricultural products at the provincial level, which is an important guide for sustainable regional agricultural development. However, this article needs more in-depth analysis. I suggest the following three aspects for improvement:

(1) Some of the content of the article lacks support from the literature, especially in the introduction. In the introduction, the authors only cite a large number of policy documents to highlight the practical significance of this study, but the scientific significance is largely not mentioned.

We noticed that there is a lengthy discussion about policy documents. Thus, we made deletions and added explanations of scientific significance.

*In the Introduction section, the* *policy documents paragraph was rephrased as followed:*

In 2022, China’s Ministry of Agriculture and Rural Affairs rolled out the “14th Five Year Plan for International Cooperation in Agriculture and Rural Affairs”, emphasising the enhancement of international collaboration in agriculture and the development of competitive advantages in agricultural trade. This focus on competitive advantage has now become a significant influencing factor in China’s agricultural cooperation endeavours [1].

*The explanation of scientific significance was added in the Introduction:*

Despite this progress, Chinese agriculture contends with challenges such as limited resources, land fragmentation, and high production costs [2]. These issues have impacted the competitiveness of China’s agricultural exports, which have revealed a comparative disadvantage [3,4], hindering the export growth and sustainable development of the sector [5]. In light of these circumstances, it is imperative to assess the current situation, identify the reasons, and understand the factors influencing the competitiveness of China’s agricultural product exports. This will aid in shaping a high-quality development model for China’s agricultural trade within the existing development framework.

(2) The authors should discuss the key findings obtained from the study.

We thank the reviewer for raising this important point. We have made some additions and deletions to the discussion on the research conclusions.

*1)In the 5.Network structure characteristics and evolution analysis section, the following phrase was added:*

It is clear that provinces selectively opt for specific export markets for their agricultural products to cultivate a competitive advantage. However, this advantage in competitiveness falls under 4% across the 174 countries (regions) analysed in this paper. Thus, the competitiveness of Chinese provincial agricultural exports is essentially minimal.

*The following phrase was deleted:*

The process of choosing export markets for agricultural products can create a degree of exclusivity, establishing a competitive advantage and potentially reducing or avoiding competition among different agricultural products in the same international market. As a result, it will facilitate the establishment of a competitive edge in global markets and serve as a demonstration base for agricultural exports from the province.

*So, the first conclusion is:*

Firstly, there are significant opportunities to improve the competitive edge of agricultural product exports across various Chinese provinces.

*2) In the 5.Network structure characteristics and evolution analysis section, the following phrase was added:*

However, the yearly rankings of the top 5 agricultural products show variability and instability across provinces, with only Guangdong Province consistently showing a competitive edge.

*So, the second conclusion is:*

Secondly, with the exception of Guangdong Province, the types of competitively robust agricultural products exported by each province are inconsistent.

*3)Based on the research findings of the paper, the third conclusion is:*

Thirdly, international sister-city relationships can foster the development of an export competitiveness network of Chinese provincial agricultural products, whereas geographic distance may hinder this network.

*4) the fourth conclusion is:*

Lastly, the development of this competition-focused export network is also influenced by province-level and national (or regional) characteristics, and there are unique differences within these influences.

(3) Authors should enhance the condensation of the conclusion and abstract sections rather than simply repeating important findings.

We apologize for the redundancy concerning the statements in the conclusion and abstract sections of the paper. We have sorted out the article's conclusions and extracted key conclusions.

*In the conclusion and abstract section, the corresponding paragraph was rephrased as followed:*

Our analysis leads to several conclusions. Firstly, the competitive edge of agricultural exports from various Chinese provinces needs bolstering. Secondly, aside from Guangdong Province, the stability of competitively advantaged agricultural exports is uncertain in other provinces. Thirdly, the international sister-city network aids the development of Chinese provincial agricultural product export competitiveness, whereas distance hinders the formation of competitive export trade relationships. Lastly, the creation of this export competitiveness network is also influenced by provincial and national (regional) characteristics, suggesting a degree of heterogeneity.

We are grateful for the constructive comments of reviewer 2 that helped to strengthen the manuscript.

Reviewer #2: The manuscript, titled "Research on the evolution of the structure and influencing factors of the export competitiveness network of Chinese provincial agricultural products – Based on a two-mode network perspective," has examined the changes in agricultural exports and the factors that impact their export competitiveness.

This is an excellent paper, and it deals with an important and timely issue using quantitative analytical tools. They have rightly used network analysis. However, the authors must address the following issues to enhance the paper's quality, readability, and communication.

•Title of the paper: The title of the manuscript is very long. The title of an article is very important to enhance its searchability, reach the audience, engage readers, influence their thoughts, and increase the probability of citations. The title is expected to be understandable to people, even those who are not experts in the field. Therefore, to reflect the content and make it easily understandable, the title may be revised as "Export competitiveness network of Chinese provincial agricultural products: Evolution, performance, and influencing factors."

Research on the evolution of the structure and influencing factors of the export competitiveness network of Chinese provincial agricultural products – Based on a two-mode network perspective

As proposed by the reviewer, we adopted revised title as the title of our paper:

Export competitiveness network of Chinese provincial agricultural products: Evolution, performance, and influencing factors.

•Strengthen the discussion section: The authors cited other studies, mainly in the introduction and methodology section, to highlight the importance of examining an issue. They have not compared the results of the present study with other studies dealing with similar topics, such as the export competitiveness of agricultural products (for example, Long, 2020) and the influence of Belt and Road on competitive advantage (for example, Feng et al., 2020). They can also cite the results of studies on trade competitiveness and international sister cities. For example, the manuscript has cited the study by Ramasamy and Cremer (1998), who analyzed the economic role of international sister-city relationships between New Zealand and Asia. Readers and policymakers will appreciate it if the authors cite the results of such studies. Thoughtful comparison always enhances the quality and impact of our research.

We apologize for not having compared the results of the present study with other studies dealing with similar topics. As suggested, we *added literature comparison and* rephrased the respective sentences in the corresponding section.

*In the 2. Literature review section,* *the paragraph was rephrased as followed:*

In summary, while current literature provides extensive theoretical and empirical research on the competitive advantage of agricultural trade and trade networks, it lacks a focused, detailed look at the export competitiveness of agricultural products from individual Chinese provinces. Some studies have examined the export competitiveness of Chinese agricultural products [5], but none have assessed the influencing factors and evolutionary patterns of such competitiveness via a network perspective. This gap leaves us without a comprehensive understanding of provincial agricultural product export networks.

*In the 4.1 Network construction section, the second paragraph was rephrased as followed:*

Feng et al. [52] primarily researched RCA networks along the Belt and Road without categorising the products. These fall under one-mode networks. We examine the export competitiveness network of different agricultural products from a more granular, provincial viewpoint, which is classified as a two-mode network. This micro two-mode network is particularly suitable for analysing the export competitiveness of Chinese provinces’ agricultural products.

We are thankful to reviewer 3 for his valuable comments that helped to profoundly improve the manuscript.

Reviewer #3: The paper has assessed the competitive advantage position of provincial agricultural product exports in the international market and investigated the influencing factors of the competitiveness network of provincial agricultural product exports.

1. Does the citation format of the literature meet the requirements of the journal? The author needs to confirm it again.

We thank the reviewer for raising this important point. Based on the requirements and specifications of the journals regarding formatting, we used endnote software to fully adjust the formatting of references.

2. In introduction, the main research problems and research contributions of this study should be clearly defined.

*As suggested, we rephrased the research problems and research contributions of this study in the Introduction section.*

The innovations in this study are primarily seen in several aspects. First, using provincial agricultural product export data, we categorise agricultural product types based on the 4-digit Harmonized System (HS). This allows for a detailed exploration of the current status and issues concerning the global reach of Chinese provincial agricultural products. Second, applying a two-mode network perspective and an enhanced revealed comparative advantage index, we innovatively construct an export competitiveness network of provincial agricultural products. This provides insight into the network’s evolution, growth, and the current state of provincial export competitiveness for agricultural products in China. Third, by using the Exponential Random Graph Model (ERGM), we investigate the factors influencing the development of the export competitiveness network of provincial agricultural product and offer sound policy suggestions for nurturing competitive advantages in provincial agricultural products.

3. It is suggest the authors to add a discussion on the application research of the dual mode network.

*We thank the reviewer for raising this important point.* *As suggested, we added a discussion on the application research of the two-mode network.*

**Research on the two-mode network**

Initial research into two-mode networks emphasised clustering, density, and centrality metrics [23]. Since then, indicators of structural features and empirical econometric models have been developed [24,25]. By adopting dynamic thinking, researchers have evolved the study of two-mode networks from a static to a dynamic perspective [26]. Various practical applications of two-mode networks such as firm-city networks [27,28], debt-bonding networks between non-financial and financial listed companies [29], networks mapping Southern women’s activity participation [30], stakeholder and cost management networks [8], and fund-stock networks [31], have been thoroughly explored. Such studies typically encompass the conversion of a two-mode network into a one-mode network, the structural assessment of a two-mode network, and the analysis of factors influencing a two-mode network.

4. The figures from this manuscript are not clear. Please ask the authors to resolve this issue.

We apologize that the graphs are blurry in the paper. The figures in the paper are as follows:

**Fig. 1. A schematic diagram of the construction of a two-mode network for provincial agricultural exports.**

(a)

(b)

**Fig 2. The export competitiveness networks of the top five provincial agricultural products in 2015 (a) and 2022 (b).** (data source: Chinese customs data). Note: The red nodes represent provincial agricultural products, and their size is proportional to the degree; the purple nodes represent countries or regions. The edges represent TCA values, and their widths are positively correlated with the TCA values.

(a)

(b)

**Fig 3. The number of agricultural products with competitive advantages that Chinese provinces exported in 2015 (a) and 2022 (b).** (data source: Chinese customs data).

5. Does Guangxi produce coconut? Please ask the authors to confirm it.

As suggested, we went further and checked the data. In a report on the development of the coconut industry, it is noted that coconuts are mainly distributed in Hainan, Yunnan, Guangdong, Guangxi, Fujian, Taiwan and other provinces and regions of China. Among them, Hainan is the main production area. We looked further into Chinese customs data. In 2022, a total of 11 provinces in China exported coconuts, and only Guangxi province exported coconuts to the United States. Therefore, Guangxi produces coconut.

6. The models required for this study should be placed in one section

We thank the reviewers for this suggestion. Two parts of this paper include formulas. One, in Chapter 4, analyses the network by constructing the network and the network metrics. This part of the formulas aims to perform a network structure analysis to describe the development process of constructing the network. Secondly, the formulas are involved in the model construction part of Chapter Five. This part is based on Chapter 4. They are differentiated. Generally speaking, the research paradigm using network analysis methodology includes network structure analysis and empirical regression analysis, with different research contents and perspectives. After much deliberation, placing the two parts of the formula in the same section is inappropriate.

7. The robust analysis should be added.

We thank the reviewer for pointing to this important methodological aspect of our paper. We have now added robust analysis in the Analysis of regression results section.

**Robustness analysis**

This paper employs lagged data and replacement regression methods for robustness testing. The corresponding regression results can be found in Table 6.

**Table 6.** Robustness test.

|  | G | | | | lnG | |
| --- | --- | --- | --- | --- | --- | --- |
|  | 2016 | 2018 | 2020 | 2022 | plm | |
| edges | -4.648^***^ | -5.153^***^ | -6.403^***^ | -4.459^***^ | lnfriendcity | 0.006^***^ |
|  | (-57.38) | (-50.52) | (-66.70) | (-56.44) |  | (4.51) |
| b1star2 | 0.179^***^ | 0.170^***^ | 0.149^***^ | 0.154^***^ | lndist | -0.651^***^ |
|  | (59.67) | (56.67) | (74.50) | (77.00) |  | (-20.42) |
| b1star3 | -0.006^***^ | -0.005^***^ | -0.004^***^ | -0.004^***^ | lnProGDP | -0.030 |
|  | (-25.82) | (-24.95) | (-27.68) | (-25.19) |  | (-0.34) |
| Nfriendcity | 1.871^***^ | 1.750^***^ | 1.765^***^ | 1.892^***^ | lnAFAHF | 0.001 |
|  | (8.54) | (9.56) | (10.09) | (10.87) |  | (0.02) |
| Ndist | -0.999^***^ | -0.930^***^ | -1.007^***^ | -1.042^***^ | lnExport | 0.084^***^ |
|  | (-6.17) | (-6.74) | (-7.40) | (-7.78) |  | (2.69) |
| ProGDP | 0.010 | -0.071 | -0.003 | 0.061 | lnCougdp | -0.007 |
|  | (0.14) | (-1.08) | (-0.04) | (0.97) |  | (-0.21) |
| AFAHF | 0.053 | 0.106^***^ | 0.084^**^ | 0.037 | lnTradeF | -0.004 |
|  | (1.47) | (3.31) | (2.63) | (1.42) |  | (-0.20) |
| Export | 0.121^***^ | 0.124^***^ | 0.107^***^ | 0.083^***^ | lnMonetaryF | 0.003 |
|  | (5.26) | (5.39) | (4.12) | (3.46) |  | (0.18) |
| CouGDP | -0.058^***^ | -0.467^***^ | -0.208^***^ | 0.098^***^ | lnPeople | 0.834^*^ |
|  | (-3.41) | (-25.94) | (-13.87) | (6.53) |  | (1.78) |
| TradeF | 0.031^***^ | 0.022^***^ | -0.008^***^ | 0.002^**^ | Constant | 18.687 |
|  | (29.45) | (21.73) | (-12.38) | (2.85) |  | (1.20) |
| MonetaryF | -0.029^***^ | 0.004^***^ | 0.029^***^ | 0.007^***^ | FE | Yes |
|  | (-29.06) | (3.97) | (28.32) | (8.42) | Observations | 107,846 |
| People | 0.054^**^ | 0.004 | 0.358^***^ | -0.293^***^ | R2 | 0.084 |
|  | (2.70) | (0.20) | (19.89) | (-17.24) | Adjusted R2 | 0.082 |
| AIC | 115328.207 | 123294.792 | 128068.574 | 139770.993 | Residual S. E. | 2.280 (df = 107643) |
| BIC | 115459.298 | 123426.433 | 128199.926 | 139903.760 | F Statistic | 48.778^***^  (df = 202; 107643) |

Note: *, **, and *** are significant at 10%, 5%, and 1% significance levels, respectively. The regression coefficients in parentheses correspond to the estimated t-values.

Regression analyses of Chinese provincial agricultural products’ export competitiveness network for 2016–2022, based on lagged-by-one-period external covariate network and node attribute indicators, demonstrate that the pure structural effects and external covariate network coefficients align closely with the benchmark regression results. Besides, export attribute variable coefficients also match the benchmark regression results. However, the coefficients of AFAHF diverge from these results. Hence, the regression utilising the lagged term provides robust outcomes.

The fixed effects model was reused for robustness testing. The results demonstrate that the coefficients of key variables, namely international sister-city (lnfriendcity), bilateral distance (lndist), and provincial export (lnExport), align with the benchmark regression outcomes, both in terms of positivity, negativity, and significance. Thus, the findings remain robust even after altering the regression method.

8. More robust analysis should be provided.

As proposed by the reviewer, we added more analysis in the results and discussion.

*We rephrased the sentences in the analysis of the regression coefficients of the province nodes attributes.* *The paragraph '* *Secondly, concerning node attribute variables' was changed as followed:*

Secondly, concerning node attribute variables, positive regression coefficients have been observed for provincial node attributes like AFAHF and export over time. This indicates that both AFAHF and export significantly contribute to establishing the provincial agricultural products’ export competitiveness network.

The provincial AFAHF measures the scope and outcomes of agricultural, forestry, livestock, and fishery production. A higher value suggests a propensity for a province to develop competitive advantages in its agricultural products, indicating heightened market influence and competitiveness. In recent years, there has been a surge in provincial exports, leading to the establishment of a trade network for agricultural products in each province. This network has facilitated access to international markets, increasing the global market share of these products and fostering competitive advantages.

*The paragraph '* *The effect of national (or regional) attributes ' was changed as followed:*

The effect of national (or regional) attributes on the competitive advantage of China’s provincial agricultural exports remains unclear. The unpredictability of CouGDP’s positive and negative coefficients could relate to the possibility that financially developed countries or regions are more probable recipients of agricultural exports from China’s various provinces. This scenario makes it difficult for each province’s agricultural exports to form a competitive edge in their target export markets, meaning CouGDP’s influence on their competitive advantage is statistically insignificant. Furthermore, the trade freedom (TradeF) coefficient generally shows a significantly positive value. This implies that as a significant economic freedom index indicator, a higher value equates to fewer tariffs and tariff barriers, thus lowering trade costs and increasing Chinese agricultural products’ accessibility in the host market. This might positively impact the selection of agricultural product export markets from Chinese provinces. The importance of promoting high-level agricultural liberalisation to ensure seamless exports from China has risen. Therefore, these findings offer partial support to H2.

*The following has been added to the paragraph 'However, there are differences in how provincial and national (regional) node attributes':*

However, there are differences in how provincial and national (regional) node attributes impact the formation of competitive advantages in agricultural product exports across these regions. The GDP of a country or region positively influences the formation of a competitive advantage in agricultural product exports in eastern provinces, but it has a negative effect in the central and western provinces. The motive behind this is that agricultural exports from the eastern provinces benefit from lower transportation costs; hence, they have more options for their export destinations. Given that developed countries generally have a higher demand for agricultural products, eastern provinces’ agricultural products are more likely to be exported to these markets, thereby facilitating a competitive edge.

In contrast, the central and western provinces may export agricultural products to nearby countries due to transport cost limitations. Trade freedom negatively impacts eastern provinces’ competitive advantage in agricultural product exports but benefits western provinces. Despite fostering product exports, trade freedom hinders eastern provinces from forming competitive agricultural product relationships due to an over-supply in high-freedom trade markets.

*The following has been added to the paragraph 'The coefficient for the international sister-city network':*

This reveals that the friendly relations between Chinese provinces and countries (regions) along the Belt and Road are not entirely mirrored in the provinces’ agricultural export competitiveness.

Ultimately, the establishment of competitive agricultural export relationships by Chinese provinces along the Belt and Road route is also shaped by contractual relationships, reducing the influence of sister-city relations.

9. Finally, this manuscript needs careful editing by someone with expertise in technical English editing.

As proposed by the reviewer and in order to avoid grammatical or typographical errors, we've asked native English speakers to do the linguistic checking and polishing about the final manuscript.

Reviewer #4: Dear Authors,

Your research on the evolution of the export competitiveness network of Chinese provincial agricultural products and its influencing factors offers valuable insights into a critical area of agricultural economics and international trade. After a thorough review, I would like to provide some constructive feedback to enhance the clarity, depth, and impact of your work.

1. Originality and Significance:

Your study makes a significant contribution by using export customs data from 2015 to 2022 to analyze the competitive network of agricultural exports across various Chinese provinces. The focus on the dynamic evolution of this network and the systematic analysis of influencing factors is commendable. However, it would be beneficial to more explicitly state how this work builds upon and diverges from existing literature in the field. Highlighting the novel aspects of your research will underscore its contribution to academic and practical knowledge.

*As suggested, we rephrased the research problems and research contributions of this study in the third paragraph of Introduction section.*

The innovations in this study are primarily seen in several aspects. First, using provincial agricultural product export data, we categorise agricultural product types based on the 4-digit Harmonized System (HS). This allows for a detailed exploration of the current status and issues concerning the global reach of Chinese provincial agricultural products. Second, applying a two-mode network perspective and an enhanced revealed comparative advantage index, we innovatively construct an export competitiveness network of provincial agricultural products. This provides insight into the network’s evolution, growth, and the current state of provincial export competitiveness for agricultural products in China. Third, by using the Exponential Random Graph Model (ERGM), we investigate the factors influencing the development of the export competitiveness network of provincial agricultural product and offer sound policy suggestions for nurturing competitive advantages in provincial agricultural products.

*In the 2. Literature review section, the last two paragraphs were rephrased as followed:*

In summary, while current literature provides extensive theoretical and empirical research on the competitive advantage of agricultural trade and trade networks, it lacks a focused, detailed look at the export competitiveness of agricultural products from individual Chinese provinces. Some studies have examined the export competitiveness of Chinese agricultural products [5], but none have assessed the influencing factors and evolutionary patterns of such competitiveness via a network perspective. This gap leaves us without a comprehensive understanding of provincial agricultural product export networks. As a result, we struggle to analyse provincial export strengths and weaknesses and to develop high-quality strategies for agricultural product exports in different provinces. With these considerations in mind, this article aims to study the high-quality development of agricultural trade in China under the lens of the “dual circulation” of international and domestic markets. By using provincial export data, it aims to establish a competitive network for agricultural product exports to various countries and regions. The objective is to analyse the current competitive advantages, challenges, and issues faced by agricultural product exports from different provinces within the international marketplace.

This thesis extends the existing literature by contributing to two vital areas. Academically, it addressed the evolution of the Chinese provincial agricultural products’ export competitiveness network and its driving factors, adopting a two-mode network perspective. The study takes a more nuanced approach to examine the high-quality growth of China’s agriculture, offering innovative research methodology and perspective. On the practical side, it substantiates policy-making around the export of agricultural products from China’s various provinces. Furthermore, it aids their assimilation into the high-quality development of the Belt and Road Initiative, providing an advanced scientific foundation.

2. Theoretical Framework:

The paper could benefit from a clearer articulation of the theoretical framework guiding the analysis. While the empirical approach is robust, integrating a theoretical perspective on networks in international trade or theories of competitive advantage could provide a stronger foundation for interpreting the results. This addition would also help in situating your work within broader academic discussions.

We apologize for not having a theoretical framework in the paper. As suggested, we conducted a theoretical analysis and formulated hypotheses based on the three factors explored.

*We added the third part: ‘3. Theoretical analysis and hypothesis’:*

**Theoretical analysis and hypothesis**

**Network pure structural effect**

Network relationships with inherent interdependencies foster the creation of additional relationships, culminating in a network. This phenomenon is referred to as the “network pure structural effect” [32,33]. Pure structural metrics such as edges, 2-stars, 3-stars, 3-paths, and 4-loops effectively conform to the Markov assumption [25]. The star structure is regarded as a “convergence” effect or a “rich get richer” effect [34], which increases the probability of connected entities establishing more distant connections [35]. In other words, nodes with a high degree of network neutrality are likely to form more connections. For Chinese provinces, this implies they are inclined to form additional relationships with other countries and regions worldwide after establishing competitive export trade relationships for agricultural goods. From these observations, we propose the following hypothesis.

H1: The star effect of the export competitiveness network of provincial agricultural products contributes to the formation of network relationships.

**Node attributes effect**

Individual qualities play an important role in forming network relationships [36]. The principle of homophily suggests that individuals are more likely to form relationships with those who share similar attributes, thereby influencing the probability of network connectivity [37,38]. Consequently, this principle substantiates the impact of individual attributes on network relationship formation in social networks.

Economic attributes impact trade behaviour and the overall network structure [39]. For instance, a country’s openness and reciprocity can affect the formation of wheat trade relations [40]. Likewise, the intensity of technological R&D and a province’s technological openness are key determinants in the evolution of interprovincial trade networks [41]. From these observations, the following hypothesis is formed.

H2: Provinces and economies with larger populations and higher levels of economic development are more inclined to establish competitive export trade relationships for agricultural products.

**External covariate effect**

Sociologists argue that an individual’s social capital comprises the resources available within their social relationships [42], including the sum of existing and potential resources in their social network [43]. This social capital can foster more collaboration and provide beneficial resources to an individual [44]. It is fundamentally defined by the network of social relationships [45] and can be considered an investment in such relationships [46]. Economically, social capital is understood as broader trust and norms [47].

In the realm of local governmental diplomacy, international sister-city relationships enable cities to accumulate social capital in their host country. This enhances the knowledge of stock about foreign markets [48], assisting not only in grasping trade and investment opportunities but also in reinforcing identity and trust. Furthermore, it reduces trade disputes and friction, influencing the establishment and robustness of trade network relationships between cities and countries. However, bilateral geographical distance elevates the cost of trade, subsequently diminishing the establishment of competitively advantageous trading relationships. Based on these considerations, this study puts forth the following hypotheses:

H3: International sister-city relations can contribute to the formation of relationships in the export competitiveness network of Chinese provincial agricultural products.

H4: Bilateral geographical distance may be an impediment to the establishment of relationships in the export competitiveness network of Chinese provincial agricultural products.

3. Methodology:

The methodology section is well-developed, leveraging export customs data to construct a network analysis of provincial agricultural product competitiveness. However, details on the specific network analysis techniques and statistical methods used would enhance the reader's understanding and the reproducibility of the study. Additionally, discussing any limitations of your methodological approach would lend credibility to your findings.

We thank the reviewer for raising this important point. As suggested, we added the respective sentences in the methods section.

*In the 4. Research design section, the following sentence was added:*

The article, using Chinese customs data, examines the dynamic evolution and competitive edge of agricultural exports across different Chinese provinces. Complex network analysis techniques are leveraged to build competitiveness networks and evaluate network structure metrics for provincial agricultural products. Complex networks, a rising area in complexity research, explore the properties of intricate systems by abstracting them into networks of interconnected nodes and edges [49]. It is an expansion of social network analysis that illustrates node heterogeneity, structural complexity, and dynamic variability in network patterns via topological indicators like loop numbers, node strength, average clustering coefficient, and betweenness centrality [50].

*In the 6.1 Construction of ERGM and variable explanation section, the following sentence was added:*

The ERGM is a crucial empirical regression model utilised in complex network analysis. It helps explain the characteristics of network structure and the factors that influence its creation. The ERGM views the whole network structure as a combination of local network effects, seeing the observed network as one possible outcome from a set of random networks [56]. The aim of the ERGM is to comprehend the formation of the entire network via its local structure [57]. It sheds light on the inception and causation of social network relationships by perceiving network connections as endogenous and interdependent [35]. The ERGM is commonly used as a statistical analysis tool for both one-mode [39,58,59] and two-mode networks [25,60,61].

*In the7.3. Limitations section, a descriptive paragraph on the limitations of the research methodology was added:*

**Limitations**

ERGM is primarily used to empirically analyse the influence of relationship establishment. However, future research should focus on empirically analysing the impact of the relationship strength, specifically exploring the factors that influence the strength of relationships within the export competitiveness network of Chinese provincial agricultural products.

4. Results and Discussion:

Your findings regarding the increasing competitiveness of provincial agricultural exports, the need for enhanced competitive advantages, the role of international sister city relationships, and the influence of agricultural output, exports, and trade freedom indexes are intriguing. It would be valuable to deepen the discussion on how these factors interplay and their implications for policy and practice. Exploring the heterogeneity in the effects of node attributes across different provincial regions and export destinations in more detail could provide nuanced insights into strategic planning for export competitiveness.

As proposed by the reviewer, we added more analysis in the results and discussion.

*We rephrased the sentences in the analysis of the regression coefficients of the province nodes attributes.* *The paragraph '* *Secondly, concerning node attribute variables' was changed as followed:*

Secondly, concerning node attribute variables, positive regression coefficients have been observed for provincial node attributes like AFAHF and export over time. This indicates that both AFAHF and export significantly contribute to establishing the provincial agricultural products’ export competitiveness network.

The provincial AFAHF measures the scope and outcomes of agricultural, forestry, livestock, and fishery production. A higher value suggests a propensity for a province to develop competitive advantages in its agricultural products, indicating heightened market influence and competitiveness. In recent years, there has been a surge in provincial exports, leading to the establishment of a trade network for agricultural products in each province. This network has facilitated access to international markets, increasing the global market share of these products and fostering competitive advantages.

*The paragraph '* *The effect of national (or regional) attributes ' was changed as followed:*

The effect of national (or regional) attributes on the competitive advantage of China’s provincial agricultural exports remains unclear. The unpredictability of CouGDP’s positive and negative coefficients could relate to the possibility that financially developed countries or regions are more probable recipients of agricultural exports from China’s various provinces. This scenario makes it difficult for each province’s agricultural exports to form a competitive edge in their target export markets, meaning CouGDP’s influence on their competitive advantage is statistically insignificant. Furthermore, the trade freedom (TradeF) coefficient generally shows a significantly positive value. This implies that as a significant economic freedom index indicator, a higher value equates to fewer tariffs and tariff barriers, thus lowering trade costs and increasing Chinese agricultural products’ accessibility in the host market. This might positively impact the selection of agricultural product export markets from Chinese provinces. The importance of promoting high-level agricultural liberalisation to ensure seamless exports from China has risen. Therefore, these findings offer partial support to H2.

*The following has been added to the paragraph 'However, there are differences in how provincial and national (regional) node attributes':*

However, there are differences in how provincial and national (regional) node attributes impact the formation of competitive advantages in agricultural product exports across these regions. The GDP of a country or region positively influences the formation of a competitive advantage in agricultural product exports in eastern provinces, but it has a negative effect in the central and western provinces. The motive behind this is that agricultural exports from the eastern provinces benefit from lower transportation costs; hence, they have more options for their export destinations. Given that developed countries generally have a higher demand for agricultural products, eastern provinces’ agricultural products are more likely to be exported to these markets, thereby facilitating a competitive edge.

In contrast, the central and western provinces may export agricultural products to nearby countries due to transport cost limitations. Trade freedom negatively impacts eastern provinces’ competitive advantage in agricultural product exports but benefits western provinces. Despite fostering product exports, trade freedom hinders eastern provinces from forming competitive agricultural product relationships due to an over-supply in high-freedom trade markets.

*The following has been added to the paragraph 'The coefficient for the international sister-city network':*

This reveals that the friendly relations between Chinese provinces and countries (regions) along the Belt and Road are not entirely mirrored in the provinces’ agricultural export competitiveness.

Ultimately, the establishment of competitive agricultural export relationships by Chinese provinces along the Belt and Road route is also shaped by contractual relationships, reducing the influence of sister-city relations.

5. Policy Implications and Recommendations:

The policy recommendations provided are a strong point of the paper, particularly the emphasis on cultivating distinctive and globally competitive agricultural products and leveraging the Belt and Road initiative. Expanding on how these strategies can be implemented at both the provincial and national levels, considering the varied economic and agricultural contexts across China, would make the recommendations more actionable.

We thank the reviewer for suggesting how these strategies can be implemented at the provincial and national levels. In the 7.2. Policy implications section, we adjusted the policy recommendation sentence.

*The paragraph on policy implications at the provincial level was changed as followed:*

(1) The improvement and development of traditional advantageous agricultural products aim to boost their international competitiveness and influence in various provinces of China. To achieve this, we will support standardised, large-scale, and modernised production to enhance the quality of these products. Recognising the economic and agricultural diversity across China, we aim to create distinct and competitive agricultural products in each province to avoid market saturation. By nurturing high-quality, globally competitive agricultural products, we will ensure every province can successfully penetrate the international market with its unique agricultural offerings.

*The paragraph on policy implications at the national level was changed as followed:*

(2) The Belt and Road Initiative presents an excellent opportunity to promote synergy in the development of high-quality agricultural products from various Chinese provinces in the global market. Trade cooperation with countries or regions along the Belt and Road, coupled with reliable logistics and infrastructure for agricultural goods, will provide an effective conduit for these exceptional products to reach international markets. Simultaneously, we must lay out differentiated plans for various provinces’ agricultural products in international markets to prevent market saturation and promote the harmonious advancement of agricultural exports from different provinces.

6. Writing Quality and Organization:

Overall, the manuscript is well-organized and clearly written. Ensuring consistency in terminology and further proofreading to catch any grammatical or typographical errors would polish the final manuscript. It may also be helpful to include more visual aids, such as charts or graphs, to illustrate key findings and trends.

As proposed by the reviewer and in order to avoid grammatical or typographical errors, we've asked native English speakers to do the linguistic checking and polishing about the final manuscript.

*As suggested, we added Figure 3 to illustrates the number of agricultural products with competitive advantages that Chinese provinces exported in 2015 and 2022.*

(a)

(b)

**Fig 3. The number of agricultural products with competitive advantages that Chinese provinces exported in 2015 (a) and 2022 (b).** (data source: Chinese customs data).

In conclusion, your manuscript provides a valuable contribution to the field of agricultural economics and international trade. With the suggested revisions, I believe the paper will offer stronger insights and clearer guidance for researchers, policymakers, and practitioners interested in enhancing the export competitiveness of Chinese provincial agricultural products. I look forward to seeing the revised manuscript and believe it has the potential to make a significant impact.
