# Supplementary material for: Export competitiveness network of Chinese provincial agricultural products: E volution, performance, and influencing factors
Source: PLoS One. 2025 Jan 16;20(1):e0316088. doi: 10.1371/journal.pone.0316088 (PMC11737858; doi:10.1371/journal.pone.0316088)
Supplement: Attachment — Submitted filename: Response to Reviewers.docx [file pone.0316088.s006.docx]

We are thankful to reviewer 3 and reviewer 4 for their valuable comments that helped to improve the manuscript once again.

Reviewer #3: The revision has been well improved based on the comments of the reviewers. But, the manuscript still needs some minor improvements based on following comments.

1. The understanding of the research innovations from the authors is biased. The three research innovations mentioned in this paper seem to be the main focus of this study.

We thank the reviewer for raising this important point. We rephrased the research innovations in the Introduction section.

(1) Most existing literature studies the export competitiveness of Chinese agricultural products from a national perspective. This paper, however, connects Chinese provinces with exported agricultural products and examines the international competitiveness of Chinese provincial agricultural products from a more micro perspective.(2) By applying a two-mode network perspective and an enhanced revealed comparative advantage index, this paper innovatively constructs an export competitiveness network of provincial agricultural products, which further enriches the research paradigm and methodology on the trade relationship. (3) Using the Exponential Random Graph Model (ERGM), this paper investigates the factors influencing the development of the export competitiveness network of provincial agricultural products from a comprehensive perspective. Compared to the traditional linear regression model, ERGM can better track the endogenous dependencies in forming the export competitiveness network, a task the conventional regression model cannot accomplish.

2. The current literature lacks a focused, detailed look at the export competitiveness of agricultural products from individual Chinese provinces. But, the authors did not mention the necessity of this research in the introduction.

We apologize for not mentioning the necessity of the export competitiveness of agricultural products from individual Chinese provinces. As suggested, we added a paragraph in the Introduction section.

The main objective of this study is to investigate the status and influence of Chinese provincial export competitiveness for agricultural products from the perspective of complex networks. The study aims to explore the evolution of the export competitiveness network of Chinese provincial agricultural products and to identify the factors affecting the competitive advantages of agricultural exports at the provincial level. The current literature lacks a focused and detailed exploration of the export competitiveness of agricultural products from Chinese provinces. This study fills this gap by conducting a comprehensive analysis from micro and macro perspectives.

3. In Empirical analysis section, there are still relatively little comparative analysis with other methods and literature.

We thank the reviewer for raising this important point. We have made some additions to the comparative analysis with other methods and literature.

In contrast to traditional linear regression models, such as those in Zhou and Tong [4] and Huyen and Bang [10], ERGM does not exclude the endogeneity problem. Additionally, the model incorporates three different levels of explanatory variables and is capable of identifying key influences as comprehensively as possible [39]. Therefore, ERGM is widely used as a statistical analysis tool for both one-mode [58,59] and two-mode networks [25,60,61].

4. The main conclusions and policy implications do not seem to correspond one-to-one. Meantime, the policy implications should have specific implementation entities.

As proposed by the reviewer, we adjusted the policy recommendations based on our conclusions one by one and indicated the implementing entity for each policy.

*The first policy implication corresponds to the first conclusion.*

(1) Enhancing and developing agricultural products with traditional advantages in China's provinces, strengthening cooperation with research institutes and relevant enterprises, and cultivating distinctive and high-quality varieties will help increase their international competitiveness and influence. Specifically, the provincial government can improve the quality of agricultural products through standardized, large-scale, and modernized production models and create distinctive and competitive agricultural products in each province based on economic and agricultural diversity.

*The second policy implication corresponds to the second conclusion.*

(2) Provincial governments should actively create agricultural brands and form a group of competitive geographical indications for agricultural products to stabilize the superior agricultural products in each province. On the one hand, provincial governments should provide policy support and guidance to integrate modern agricultural industrial parks and advantageous specialty industrial clusters and create agricultural product brands with strong competitive advantages in the provinces. On the other hand, provincial governments can facilitate better exports of advantageous agricultural products by building stable and mature export routes.

*The third policy implication corresponds to the third conclusion.*

(3) Provincial governments must make the most of diplomatic relations at the local level to build a platform for agricultural cooperation. Leverage international sister-city relationships to create new possibilities for bilateral economic and trade cooperation and to enhance the competitive advantage of provincial agricultural exports in friendly countries. Encourage interactions and collaborations between provinces and global partners to increase awareness and recognition of provincial agricultural products through bilateral cultural and economic exchanges. At the same time, special attention should be paid to exploring complementary spaces for agricultural products between Chinese provinces and countries along the Belt and Road.

*The fourth policy implication corresponds to the fourth conclusion.*

(4) Different regions should carry out reasonable regional layouts and planning for agricultural products to avoid homogenization and the situation of many but not strong. The eastern region should fully utilize its geographic location to consolidate the dominant position of agricultural products in markets such as the United States, the European Union, Japan, and South Korea, while the central and western regions should actively promote the diversification of agricultural export markets based on the CHINA RAILWAY Express and the new western land-sea corridor.

Reviewer #4: The authors have largely addressed the suggestions made by the reviewers, but there are still the following details that need attention.

1. Pay attention to formatting, such as aligning the ends of the text and keeping the charts and graphs on the same page.

We thank the reviewers for this suggestion. The latest manuscript has been set up to align the ends of the text and keep the charts and graphs on the same page.

2. Simplify the content, such as the findings need to be further refined and summarised to make them more logical and organised.

We thank the reviewer for pointing to this important issue. We rephrased the sentences in the analysis of regression results.

*The content of paragraph '* *In terms of pure structural effects ' and '* *The 2-star with a positive coefficient significantly fosters ' has been simplified based on internal logic.*

The 2-star with a positive coefficient significantly fosters export competitiveness for agricultural products in the global market, indicating a “rich get richer” effect. This means that competitive export relationships are continually expanding. In situations where such relationships are already in place, Chinese provincial agricultural products tend to form more. This paper can, therefore, confirm that the model’s results verify H1. Simultaneously, the 3-star coefficient shows a significantly negative value. As trade costs rise with the addition of too many competitive export relationships for agricultural products, provinces will avoid forming numerous such relationships.

Social network analysis typically features Markov models with convergence, characterized by positive 2-star coefficients and negative 3-star coefficients [25]. A positive 2-star coefficient suggests a propensity for forming new friendships, while a negative 3-star coefficient implies potential drawbacks from establishing and sustaining a large number of friendships, indicating a “ceiling” on maintained friendships [66].China has emphasized the importance of promoting high-quality agricultural development, paying more attention to expanding favorable agricultural products in international markets, and creating new competitive advantages in exporting specialty agricultural products.

*The paragraph of '* *Secondly, concerning node attribute variables ' was rephased as followed:*

AFAHF and export significantly contribute to establishing the provincial agricultural products’ export competitiveness network. A higher provincial AFAHF suggests a propensity for a province to develop competitive advantages in its agricultural products. Provincial export establishes a channel for agricultural products in each province.

*The paragraph of '* *The effect of national (or regional) attributes on the competitive advantage ' was rephased as followed:*

CouGDP's influence on the competitive advantage of provincial agricultural exports is statistically insignificant. Financially developed countries or regions are more probable recipients of agricultural exports from China's various provinces. This scenario makes it difficult for each province's agricultural exports to form a competitive edge in the financially developed export markets.

*The part '* *China’s varying economic development and openness across its eastern, central, and western regions can lead to different factors influencing the competitive advantages of agricultural exports in each province. ' was deleted.*

*The paragraph of '* *The regression results in Table 7 indicate that ' was rephased as followed:*

The regression results in Table 7 indicate that the formation of competitive advantage for provincial agricultural product exports is influenced uniformly by structural effects and covariate networks. Due to the advanced economy in the eastern and central provinces, where the sister-city relationship has a limited role in boosting their agricultural exports’ competitiveness, the eastern and central provinces generally have lower coefficients than the western provinces. These findings emphasize the importance of considering the provincial’s economic development and openness when exploring the actors influencing the competitive advantages of agricultural exports.

*The paragraph '* *The coefficient for the international sister-city network ' and '* *Ultimately, the establishment of competitive agricultural export relationships ' has been merged as one paragraph based on the content and internal logic.*

The international sister-city network shows a significant positive relationship within both categories. However, this relationship is less pronounced for countries along the Belt and Road. This result reveals that the friendly relations between Chinese provinces and countries (regions) along the Belt and Road are not entirely mirrored in the provinces’ agricultural export competitiveness. Ultimately, the establishment of competitive agricultural export relationships by Chinese provinces along the Belt and Road route is also shaped by contractual relationships, reducing the influence of sister-city relations. Most countries along the Belt and Road possess natural geographical advantages, substantial economic development benefits, and a strong reliance on foreign trade. Thus, the establishment of sister-city relationships holds the potential to enhance cooperation and development further.

3. describe in detail the construction process of the agricultural export competitiveness network, including the definition of network nodes and edges, data processing methods and other content.

We apologize for not describing in detail the construction process of the agricultural export competitiveness network in the paper. As suggested, we have made some additions.

*The part '* *The network comprises two types of nodes ' was changed as followed:*

The network comprises two types of nodes: one is a composite node encompassing a province and a specific type of agricultural product, meaning each node corresponds to a province and the agricultural product it exports. For example, if Guangdong Province exports Vegetable Oil, then Guangdong-Vegetable Oil constitutes a node. This paper identified a total of 189 agricultural product export categories classified at the HS 4-digit level. The theoretical maximum number of nodes in this type is 189 x 31, combining 31 provinces. The other type of node represents the export destination, signifying a country or region. The edges of the two-mode network of provincial agricultural product exports are the trade relationships established between the province's agricultural products and countries.

*The paragraph '* *Feng et al. [52] primarily researched RCA networks ' was added a sentence:*

Based on the trade volume of various agricultural products exported from Chinese provinces to different countries, the TCA is constructed as follows:

4. carefully proofread and revise the language of the paper to ensure smooth and accurate language expression and avoid grammatical errors and spelling mistakes.

*As suggested, we checked the whole manuscript once again and corrected the misrepresentations.*
