# Supplementary material for: Export competitiveness network of Chinese provincial agricultural products: E volution, performance, and influencing factors
Source: PLoS One. 2025 Jan 16;20(1):e0316088. doi: 10.1371/journal.pone.0316088 (PMC11737858; doi:10.1371/journal.pone.0316088)
Supplement: Attachment — Submitted filename: Response to Reviewers.docx [file pone.0316088.s008.docx]

We are thankful to reviewer 4 and reviewer 5 for their valuable comments that helped to improve the manuscript.

Reviewer #4: The manuscript presents an interesting analysis of the export competitiveness network of Chinese provincial agricultural products from 2015 to 2022. The use of a two-mode network perspective and the Exponential Random Graph Model (ERGM) adds novelty to the research. However, there are a few points that need clarification and improvements to strengthen the manuscript.

1. Introduction Section:

The introduction could benefit from a more concise summary of the research gap, particularly international outlook, and the significance of studying export competitiveness at the provincial level. Clearly stating why this study is needed and how it differs from existing literature will strengthen the motivation. The authors mention three research innovations, but these could be framed more clearly in the context of the overall study goals and objectives.

We thank the reviewer for raising this important point. As suggested, we have made some additions and rephrased portions of the Introduction section.

The third paragraph in the Introduction section has been supplemented with additional sentences:

Notably, the primary competitive agricultural products in the global market include wheat from Russia, soybeans from the United States, and rice from Thailand. As a major agricultural nation, China is relatively deficient in globally competitive agricultural products.

The first sentence in the fourth paragraph was rephrased as follows:

The competitive advantage of exported products is crucial for the high-quality economic development of a country or region.

We have added a sentence in the fourth paragraph:

Due to variations in geographic location and resource endowment, China's exported agricultural products exhibit distinct geographical characteristics and imbalances.

A sentence in the fourth paragraph was rephrased as follows:

Therefore, it is essential to provide complete insights into the agricultural export advantages of each province [8] and to identify the factors that propel the effective international expansion of these advantageous agricultural products [9,10].

A sentence in the fifth paragraph was rephrased as follows:

Exploring the evolution of the export competitiveness network of Chinese provincial agricultural products and identifying the influencing factors will help promote the high-quality development of agricultural trade in China's provinces.

A sentence in the sixth paragraph was rephrased as follows:

This paper, however, connects Chinese provinces with their exported agricultural products and examines the international competitiveness of Chinese provincial agricultural products from a more micro perspective to further explain the regionalization and differentiation characteristics of competitive agricultural products across Chinese provinces.

A sentence has been added to the sixth paragraph:

This approach more effectively identifies agricultural products with competitive advantages in each province and further enriches the research paradigm and methodology regarding the trade relationship.

2. Methodology: Provide more details on the construction of the export competitiveness network. Clarify the definition of network nodes and edges, including the data sources and processing methods. This will help readers replicate the analysis. Explain the choice of ERGM over other statistical models, highlighting its advantages in capturing endogenous dependencies.

We apologize for not describing in detail the construction process of the agricultural export competitiveness network in the paper.

This paper develops the export competitiveness network of provincial agricultural products based on the two-mode network for provincial agricultural exports. These two types of networks have the same nodes but different edges. As suggested, we enhanced the description of the nodes and edges within the export competitiveness network of Chinese provincial agricultural products in the part of Network construction.

Therefore, the nodes of the export competitiveness network of Chinese provincial agricultural products include two categories: the composite nodes of agricultural products exported from Chinese provinces and the destination countries. The edges of the export competitiveness network are the TCA index with a value greater than 1.5.

As suggested, we have made some additions about data sources and processing methods in Network construction.

We added a sentence in the first paragraph of the Network construction:

The data was sourced from China Customs data, which has been available since 2015. By 2023, obtaining complete data for the year ending in 2022 may be possible. Therefore, the research sample in this paper is based on data from 2015 to 2022 to ensure consistency and coherence.

We added a sentence in the second paragraph of the Network construction:

We excluded the sample of countries with missing data on economic freedom for more than two consecutive years, resulting in 186 countries. From this group, we further excluded countries that had missing GDP data for more than two consecutive years. Ultimately, the final sample comprised 174 countries.

A sentence about why we determine the sample size based on HS 4 to classify provincial agricultural products has been added to the second paragraph of the Network construction:

The advantage of classifying agricultural products at the HS 4-digit level is that it effectively distinguishes between various agricultural products without leading to an excessively granular and diverse range. In this paper, we have excluded the Hong Kong, Macao, and Taiwan regions of China.

We summarize the reasons for selecting the ERGM based on three key points, the second of which illustrates the advantages in capturing endogenous dependencies.

(1) The ERGM is a crucial empirical regression model utilized in complex network analysis. It helps explain the characteristics of network structure and the factors that influence its formation. The ERGM views the whole network structure as a combination of local network effects, seeing the observed network as one possible outcome among a set of random networks [56]. Compared to the linear regression methods employed in Long [5] and Bojnec and Ferto [6], which only explore the factors influencing the competitiveness of agricultural exports from an external perspective, the ERGM provides insights into the formation of relationships and the underlying factors that shape them, grounded in the nature and logic of network construction.

(2) In contrast to traditional linear regression models, such as those presented by Zhou and Tong [4] and Huyen and Bang [10], ERGM does not exclude the issue of endogeneity. An essential category of explanatory variables in ERGM is network self-organization, which refers to the network structure of the explanatory variables. The underlying rationale is that network connections are both endogenous and interdependent [35]. Pre-existing network relationships significantly influence the formation of new connections within the network. Thereby, ERGM aims to comprehend the formation of the entire network through its local structure [57].

(3) Additionally, the model incorporates three different types of explanatory variables: self-organization, node attribute indicators, and external covariate network. Based on this, we can comprehensively identify critical influences [39] and promote the theoretical hypothesis regarding the formation of export competitiveness networks.

3. Empirical Analysis: Strengthen the comparative analysis with other methods and literature. Discuss how ERGM compares to traditional linear regression models in terms of explaining the development of the export competitiveness network. Provide more interpretation of the regression results, particularly the significance of various network effects and covariates. Link the findings back to the research questions and hypotheses.

In explaining the choice of ERGM over other statistical models, we have made some additions to compare ERGM with traditional linear regression models in the Construction of ERGM and variable explanation:

Compared to the linear regression methods employed in Long [5] and Bojnec and Ferto [6], which only explore the factors influencing the competitiveness of agricultural exports from an external perspective, the ERGM provides insights into the formation of relationships and the underlying factors that shape them, grounded in the nature and logic of network construction.

In contrast to traditional linear regression models, such as those presented by Zhou and Tong [4] and Huyen and Bang [10], ERGM does not exclude the issue of endogeneity. An essential category of explanatory variables in ERGM is network self-organization, which refers to the network structure of the explanatory variables. The underlying rationale is that network connections are both endogenous and interdependent [35].

We thank the reviewer for pointing to the interpretation of the regression results.

In part of Basic regression analysis, we have made some addition about the interpretation of network effects:

This means that competitive export relationships are continually expanding. As a result, the 2-star of the export competitiveness network of provincial agricultural products contributes to the formation of network relationships.

Provinces are likely to refrain from establishing numerous such relationships. Therefore, a negative 3-star coefficient indicates a “ceiling” on maintained friendships.

At the same time, an appropriate number of competitive export relationships is optimal.

In part of Basic regression analysis, we have made some deletion about the interpretation of network effects:

A positive 2-star coefficient suggests a propensity for forming new friendships, while a negative 3-star coefficient implies potential drawbacks from establishing and sustaining a large number of friendships.

In part of Basic regression analysis, we have made some addition about the interpretation of covariates:

These networks act as a valuable resource of social capital, providing natural advantages in enhancing bilateral trade, driving economic development and partnerships [67]. Specifically, by lowering language-related costs, sharing market information and consumer preferences, conducting agricultural cooperation projects, and optimizing supply chain management for agricultural products among sister cities, the sister-city networks ultimately build competitive advantages for agricultural exports. Consequently, playing the role of a sister-city platform has become a crucial strategy for many provinces and cities to promote their agricultural products.

4. Results and Discussion: The findings could be organized more logically and concisely. Avoid repetition and streamline the explanations to make them more accessible to readers. Discuss potential limitations of the study and suggestions for future research. For example, consider examining longer time series data or including additional covariates.

We thank the reviewer for pointing to this important result and discussion aspect of our study. In part of Robustness analysis, we have rephrased the sentences as follows:

The coefficients for pure structural effects and the lagged-by-one-period external covariate network are consistent with the benchmark regression results, and both pass the 1% significance test.

In part of Heterogeneity analysis, we have made some addition about the reasons for conducting heterogeneity analysis:

The influencing factors and effects may vary significantly among provinces due to differences in geographic location, resource endowment, economic development, and other factors.

Specific descriptions of regional divisions have been deleted in consideration of redundancy:

The eastern region includes provinces such as Beijing, Tianjin, Hebei, Shanghai, Jiangsu, Zhejiang, Fujian, Shandong, Guangdong, Hainan, Liaoning, Jilin, and Heilongjiang. The central region comprises Shanxi, Anhui, Jiangxi, Henan, Hubei, and Hunan, while the western region includes Inner Mongolia, Guangxi, Chongqing, Sichuan, Guizhou, Yunnan, Xizang, Shaanxi, Gansu, Qinghai, Ningxia and Xinjiang.

A sentence on regional divisions has been rephrased as follows:

This paper divides the 31 provinces into three regions-eastern, central, and western-based on their province location for the heterogeneity analysis.

In the Heterogeneity analysis section, the paragraph relating to Table 7 was changed as followed:

The results presented in Table 7 also reveal the heterogeneity in the impact of node attributes on the formation of competitive advantages in agricultural product exports across different regions. The CouGDP positively influences the competitiveness of agricultural product exports in the eastern provinces, but it has a negative effect in the central and western provinces. Agricultural exports from the eastern provinces benefit from lower transportation costs, making them more likely to be exported to developed markets, thereby facilitating their competitive edge. In contrast, the central and western provinces tend to export agricultural products to neighboring countries due to transportation cost constraints.

In the Heterogeneity analysis section, we have made some addition about the interpretation of node attribute indicators:

Since the western provinces must consider transportation costs, they prefer to export their competitive agricultural products to countries with open trade policies to minimize expenses.

In the Heterogeneity analysis section, the interpretation of international sister-city network has been rephrased as follows:

The international sister-city network shows a significant positive relationship in both categories. However, the coefficients for the international sister-city network are lower for countries along the Belt and Road. This result reveals that the friendly relations between Chinese provinces and countries along the Belt and Road are not entirely mirrored in the agricultural export competitiveness. Ultimately, the establishment of competitive agricultural export relationships by Chinese provinces along the Belt and Road is also shaped by contractual relationships, which diminish the influence of sister-city relations. Most countries along the Belt and Road possess natural geographical advantages and rely on foreign trade. Thus, there is a need for further examination into the role of international sister-city relationships in fostering competitive advantages in provincial agricultural exports to countries along the Belt and Road. Underpinned by a combination of national and local political relations, Chinese provinces find it easier to establish competitively advantageous agricultural export relationships.

As suggested, we addressed the deficiencies based on data and covariates.

Furthermore, the data in the China Customs database has been available since 2015, making the current timeframe under study relatively short. A longer span of data can enhance statistical credibility and improve the generalizability of the findings. Therefore, it is essential to consider examining longer time series data in future studies.

Moreover, while this paper incorporates three types of network structure statistics in the empirical regression analysis, some of these variables are not statistically significant. Future research will explore additional variables at the levels of Chinese provinces and countries to enhance its validity.

5. Policy Implications: The policy implications are well-aligned with the conclusions, but could be framed more concretely with specific actions provinces could take. Consider including real-world examples or case studies to illustrate the implementation of these policies.

We apologize for not elaborating policy implications concretely. As suggested, we have made some additions.

For the first implications, the following phrases were added:

For example, Shandong Province, recognized as a significant agricultural export hub in China, can emphasize its strengths in exporting vegetables and fruits. Meanwhile, Zhejiang Province can strengthen the international competitiveness of its tea industry, and Yunnan Province can focus on building its strengths in flower exports, among other initiatives.

For the second implications, the following phrases were added:

It is worthwhile to learn from the experiences of the wine industry cluster in California. California's wine sector has achieved a high level of intensification, characterized by mechanized grape harvesting, a well-developed integrated transportation system, and a global distribution network. As a result, operational efficiency and brand competitiveness have significantly improved.

For the third implications, the following phrases were added:

The approach taken by Guizhou Province in fostering friendly relations and signing an agreement for international agricultural cooperation with Luang Namtha Province in Laos is commendable and worth replicating.

6. Formatting and Language: Ensure the text is consistently formatted, with aligned ends and charts/graphs kept on the same page. Carefully proofread the manuscript to correct any grammatical errors, spelling mistakes, or unclear phrasing.

As suggested, we have aligned the ends of the manuscript.

The journal requires that graphs be uploaded separately from the manuscript. As suggested, we have placed the charts on the same page within the manuscript (the Revised Manuscript without Track Changes), which does not include the graphs.

As suggested, we thoroughly reviewed the entire manuscript, examining it word by word and correcting the grammatical errors, spelling mistakes, and unclear phrasing.

Reviewer #5: The manuscript can be considered for publication after the following questions and recommendations:

1.The researchers did not indicate what is new and distinctive in the abstract of this manuscript (compared to other previous studies).

We thank the reviewer for pointing to this important issue. We added a sentence in the Abstract:

This study contributes to the existing literature by investigating the performance and factors influencing the competitiveness of Chinese agricultural product exports at the provincial level, using a complex network perspective, and offers recommendations for improvement.

2.The authors did not specify the method used to determine the sample size.

As suggested, we have made some additions about data sources and processing methods in Network construction.

We added a sentence in the first paragraph of the Network construction:

The data was sourced from China Customs data, which has been available since 2015. By 2023, obtaining complete data for the year ending in 2022 may be possible. Therefore, the research sample in this paper is based on data from 2015 to 2022 to ensure consistency and coherence.

We added a sentence in the second paragraph of the Network construction:

We excluded the sample of countries with missing data on economic freedom for more than two consecutive years, resulting in 186 countries. From this group, we further excluded countries that had missing GDP data for more than two consecutive years. Ultimately, the final sample comprised 174 countries.

A sentence about why we determine the sample size based on HS 4 to classify provincial agricultural products has been added to the second paragraph of the Network construction:

The advantage of classifying agricultural products at the HS 4-digit level is that it effectively distinguishes between various agricultural products without leading to an excessively granular and diverse range. In this paper, we have excluded the Hong Kong, Macao, and Taiwan regions of China.

3.The idea of the manuscript is based on the exported products to determine which products have the most significant impact on the global market. Therefore, the Pareto chart will help draw a clearer picture for the reader and summarize the manuscript in one chart. The Pareto chart aims to highlight the most important products among a (usually large) group of products.

This paper examines the competitive advantages of agricultural products exported by various provinces in international markets. The focus is specifically on the agricultural products exported by these provinces. As shown in the paper, the theoretically possible combinations of province-agricultural products amount to 189 * 31 = 5,859. We have counted the data from the period between 2015 and 2022, reveals that the actual province-agricultural product combinations range from 2,764 to 3,004. Creating a Pareto chart based on such a large number of province-agricultural product combinations may not yield the desired insights. Therefore, we will concentrate on 31 provinces as the subjects of our study to investigate the competitively advantageous agricultural export relationships established by different provinces over the years. This approach aims to uncover the variations in competitive advantages among provinces in the export of agricultural products.

At present, the main provinces that have established many competitive agricultural export relationships are Shandong, Guangdong, Jiangsu, Zhejiang, and Fujian. See Figure 2.

Fig. 2. Pareto chart of the number of competitive agricultural export relationships established in each province in 2015 and 2022. (data source: Chinese Customs data)

4.The researchers did not mention why this regression method was used to analyze the results.

We thank the reviewers for this suggestion. We rephrased the first paragraph in the Research design.

Therefore, employing complex network analysis can effectively illuminate the structural evolution of the export competitiveness network of Chinese provincial agricultural products and identify the competitive agricultural products of these provinces. In contrast to traditional research methods, complex network analysis can investigate the key factors influencing agricultural export competitiveness at various levels, ultimately providing actionable recommendations to enhance the export competitiveness of these products in Chinese provinces.

We rephrased the Construction of ERGM and variable explanation as follows:

(1) The ERGM is a crucial empirical regression model utilized in complex network analysis. It helps explain the characteristics of network structure and the factors that influence its formation. The ERGM views the whole network structure as a combination of local network effects, seeing the observed network as one possible outcome among a set of random networks [56]. Compared to the linear regression methods employed in Long [5] and Bojnec and Ferto [6], which only explore the factors influencing the competitiveness of agricultural exports from an external perspective, the ERGM provides insights into the formation of relationships and the underlying factors that shape them, grounded in the nature and logic of network construction.

(2) In contrast to traditional linear regression models, such as those presented by Zhou and Tong [4] and Huyen and Bang [10], ERGM does not exclude the issue of endogeneity. An essential category of explanatory variables in ERGM is network self-organization, which refers to the network structure of the explanatory variables. The underlying rationale is that network connections are both endogenous and interdependent [35]. Pre-existing network relationships significantly influence the formation of new connections within the network. Thereby, ERGM aims to comprehend the formation of the entire network through its local structure [57].

(3) Additionally, the model incorporates three different types of explanatory variables: self-organization, node attribute indicators, and external covariate network. Based on this, we can comprehensively identify critical influences [39] and promote the theoretical hypothesis regarding the formation of export competitiveness networks.
