# Supplementary material for: Export competitiveness network of Chinese provincial agricultural products: E volution, performance, and influencing factors
Source: PLoS One. 2025 Jan 16;20(1):e0316088. doi: 10.1371/journal.pone.0316088 (PMC11737858; doi:10.1371/journal.pone.0316088)
Supplement: Attachment — Submitted filename: Response to Reviewers.docx [file pone.0316088.s009.docx]

We are thankful to reviewer 4 for him/her valuable comments that helped to improve the manuscript.

Reviewer #4:

（1） Improve the simplicity of language throughout the text.

We thank the reviewer for pointing to this important issue. We went through the whole text again and removed some redundant expressions.

A sentence in the third paragraph of the Introduction section was rephrased as follows:

These issues have impacted the competitiveness of Chines~~ea’s~~ agricultural exports, revealing ~~which have revealed~~ a comparative disadvantage [3,4] that hinders ~~both the~~ export growth and the sustainable development ~~of the sector~~ [5].

A sentence in the fourth paragraph of the Introduction section was rephrased as follows:

Secondly, it enhances competitiveness in the global market and improves ~~fosters the improvement of~~ the international economic circulation system [7].

A sentence in the fifth paragraph of the Introduction section was rephrased as follows:

~~In light of these circumstances, t~~The main objective of this study is to investigate the evolution, performance, ~~status~~ and ~~influence~~ influencing factors of the export competitiveness network of Chinese provincial ~~export competitiveness for~~ agricultural products through the perspective of complex networks. In this context, provinces refer to 27 Chinese provinces and 4 municipalities directly governed by the Central Government.

A sentence in the second paragraph of the External covariate effect section was rephrased as follows:

Furthermore, it reduces trade disputes and friction, thereby influencing the establishment and robustness of trade network relationships between cities and countries. However, bilateral geographical distance elevates the trade cost ~~of trade~~, ~~subsequently~~ diminishing the establishment of competitively advantageous ~~trading~~ trade relationships. Based on these considerations, this study puts forth the following hypotheses:

We have combined the first paragraph of the Network construction section with the first paragraph of the Research design section. The first paragraph of the Research design section was rephrased as follows:

The article utilizes data from~~, using~~ Chinese Customs ~~data,~~to examine~~s~~ the dynamic evolution and competitive edge of agricultural exports across ~~different~~ Chinese provinces to various countries and regions.  ~~The~~This data ~~was sourced from China Customs data, which~~ has been available since 2015. By 2023, it may be possible to obtaino~~btaining~~ complete data for the year ending in 2022 ~~may be possible~~. Therefore, the research sample in this paper is based on data from 2015 to 2022 to ensure consistency and coherence.

Some sentences in the sixth paragraph of the Network construction section were rephrased as follows:

${G\left( V,TCA \right)}_{t}$ is an $M*N$ matrix, where $M$ represents~~is~~ the composite nodes ~~set~~ of provinces and the agricultural products they export, and $N$ denotes~~is~~ the nodes~~set~~ of destination countries. ~~Therefore, the nodes of the export competitiveness network of Chinese provincial agricultural products include two categories: the composite nodes of agricultural products exported from Chinese provinces and the destination countries.~~

Since the first paragraph in the Construction of structural indicators section does not address the key elements, we have deleted this paragraph.

~~The export competitiveness network of provincial agricultural products is a typical two-mode network. This article primarily establishes the following structural indicators.~~

Some sentences in the first paragraph of the Network structure characteristics and evolution analysis section were rephrased as follows:

~~Table 1 presents the structural indicators of the export competitiveness network of provincial agricultural products over time.~~ The agricultural products exported from various provinces have proven ~~to be~~ competitive within expanding international markets, primarily evidenced by ~~an~~ increased ~~in~~ network edges and nodes. This suggests an overall improvement in the competitiveness of agricultural products.

A sentence in the fourth paragraph of the Network structure characteristics and evolution analysis section was rephrased as follows:

~~It is clear that~~ The provinces selectively choose specific export markets for their agricultural products to cultivate a competitive advantage. ~~However, this competitive advantage accounts for less than 4% across the 174 countries analyzed in this paper.~~

A sentence in the fifth paragraph of the Network structure characteristics and evolution analysis section was rephrased as follows:

~~Table 2 demonstrates a consistent~~ The ~~node degree among the~~ top 5~~five~~ countries in node degree perform consistently.

A sentence in the paragraph before the Fig 3 was rephrased as follows:

However, the yearly rankings of the top 5 provincial agricultural products in degrees show variability and instability in competitive edge across provinces, except for~~with only~~ Guangdong Province ~~consistently showing a competitive edge~~.

A sentence in the second paragraph of the Robustness analysis section was rephrased as follows:

Regression analyses of the export competitiveness network of Chinese provincial agricultural products from 2016 to 2022, based on a lagged-by-one-period external covariate network and node attribute indicators, demonstrate that the coefficients for pure structural effects and the lagged-by-one-period external covariate network ~~coefficients~~ closely align with the benchmark regression results,~~. The coefficients for pure structural effects and the lagged-by-one-period external covariate network are consistent with the benchmark regression results, and~~ both pass the 1% significance test. Besides, the coefficients for Export also match the benchmark regression results. Hence, the regression utilizing the lagged-by-one-period external covariate network ~~lagged term~~ provides robust outcomes.

A sentence in the first paragraph before the Table 8 was deleted since the usefulness was not evident:

~~Most countries along the Belt and Road possess natural geographical advantages and rely on foreign trade.~~

Some sentences in the second paragraph of the Policy implications section were rephrased as follows:

(1) Enhancing and developing agricultural products with traditional advantages ~~in~~ for ~~China's~~ Chinese provinces~~,~~ while strengthening collaboration with research institutes and relevant enterprises, which will cultivate distinctive and high-quality varieties~~, thereby~~ and increase~~ing~~ the~~ir~~ international competitiveness ~~and influence~~of agricultural products.

Some sentences in the second paragraph of the Limitations section were rephrased as follows:

First, ERGM is primarily used to empirically analyze the influence of relationship establishment. However, future research should focus on empirically analyzing the impact of relationship strength ~~, specifically exploring the factors that influence relationships strength within~~on the export competitiveness network of Chinese provincial agricultural products.

（2）Please use standard English descriptions.

As suggested, we checked the full text and rephrased some expressions of the manuscript.

A sentence in the sixth paragraph of the Introduction section was rephrased as follows:

This paper, however, ~~connects Chinese provinces with their exported agricultural products and~~ examines the international competitiveness of Chinese ~~provincial~~ agricultural products at the provincial level, providing insights into~~from a more micro perspective to further explain~~ the regionalization and differentiation characteristics of competitive agricultural products across Chinese provinces.

Some sentences in the second paragraph of the Research on the two-mode network section were rephrased as follows:

Although some studies have explored the export competitiveness of Chinese agricultural products [5], none have assessed the influencing factors and evolution~~evolutionary patterns~~ of ~~this~~ competitiveness from a network perspective. This gap hinders our comprehensive understanding of the export networks of provincial agricultural products ~~export networks~~.

Based on~~Using~~ provincial export data, this article~~it~~ aims to establish a competitive network for agricultural product exports to various countries and regions. The objective is to analyze the evolution, performance, and influencing factors of the export competitiveness network of Chinese provincial agricultural products~~competitive advantages, challenges, and issues faced by agricultural product exports from different provinces within the international marketplace~~.

A sentence in the second paragraph of the Node attribute effect section was rephrased as follows:

Economic attributes significantly influence trade ~~behavior~~ and the overall structure of trade networks [39]

Some sentences in the second paragraph of the Network construction section were rephrased as follows:

This paper ~~data helps~~ builds a two-mode network of Chinese provincial agricultural ~~product~~ exports. The nodes in the two-mode~~export~~ network ~~of Chinese provincial agricultural products~~ comprise~~s~~ two types. The first type ~~is a~~ are composite nodes encompassing ~~a~~ provinces and ~~a specific type of~~ agricultural products, meaning that each node corresponds to a province and the agricultural product it exports.

A sentence in the third paragraph of the Number of network nodes, edges, density, and average degree section was rephrased as follows:

The closer the index is to 1, the more connected the network becomes~~As the index approaches 1, the nodes within the network become denser, implying closer connections.~~

Some sentences in the third paragraph of the Network structure characteristics and evolution analysis section were rephrased as follows:

There is a notable potential for improving the competitive advantage of agricultural exports from Chinese provinces. While international trade networks usually exhibit high density [54], the density of ~~this study constructed an~~ the export competitiveness network ~~o~~ this study constructed ~~f~~ ~~provincial agricultural products around a defined threshold. Consequently, the network’s density~~ is remarkably low, consistently below 0.039~~, across the years studied~~. ~~Therefore, boosting the competitiveness of these exports is imperative.~~ Moreover, a consistent uptrend ~~was noted~~ in LPN, LPN(M) and LPN(N) ~~the average degree across all classifications,~~ indicate~~ing~~s an increase in ~~the number of~~ competitive~~dominant~~ agricultural export partnerships ~~initiated by Chinese provinces~~. On average, each ~~province’s~~ provincial agricultural ~~exports~~ product shows a competitive edge in merely five or six countries.

The first paragraph before Table 4 and the first paragraph after Table 4 were rephrased as follows:

This paper employed the stepwise method to perform an analysis aimed at ~~The initial step involves~~ identifying the most suitable regression model using the 2015 data ~~and the specific regression results presented in~~ . See Table 4.

The star effects of the country were removed as the strong correlation when incorporating the star effects of both node types in the ERGM. A~~The instability of the model results may be attributed to the strong correlation between the star effects of provincial agricultural products and the country in the ERGM. Consequently, it is not feasible to incorporate the pure structural effects of both node types into a single model. Simultaneously, a~~s this paper primarily investigates the competitive advantage from the perspective of provincial agricultural products, the model only incorporates the star effect of provincial agricultural products.

A sentence in the second paragraph after the Table 7 was rephrased as follows:

~~The results presented in Table 7 also reveal the heterogeneity in the~~ The impact of node attributes on forming~~the formation of~~ competitive advantages in agricultural product exports across different regions reveals the heterogeneity.

A sentence in the sixth paragraph of the Policy implications section was rephrased as follows:

(4) Different regions should carry out reasonable regional ~~layouts and~~ planning for agricultural products to avoid homogenization and the proliferation of products that lack competitive strength.
