# Supplementary material for: Export competitiveness network of Chinese provincial agricultural products: E volution, performance, and influencing factors
Source: PLoS One. 2025 Jan 16;20(1):e0316088. doi: 10.1371/journal.pone.0316088 (PMC11737858; doi:10.1371/journal.pone.0316088)
Supplement: Attachment — Submitted filename: Response to Reviewers.docx [file pone.0316088.s010.docx]

We are thankful to reviewer 4 for him/her valuable comments that helped to improve the manuscript.

Reviewer #4:

1.Throughout the manuscript, there are several long and complex sentences, particularly in the introduction and methodology sections. Simplifying the language will enhance readability.

We thank the reviewer for pointing to this important issue. We went through the whole text again and simplified some redundant sentences.

The second paragraph of the Introduction section was rephrased as follows:

In 2022, China’s Ministry of Agriculture and Rural Affairs rolled out the “14th Five-Year Plan for International Cooperation in Agriculture and Rural Affairs”~~,~~ . This plan ~~emphasizing~~ emphasized the importance of strengthening international collaboration ~~in agriculture~~ and fostering competitive advantages in agricultural trade. Focusing on competitive advantage has ~~now~~ become a significant factor in China’s agricultural cooperation endeavors [1].

The third paragraph of the Introduction section was rephrased as follows:

Despite this progress, Chinese agriculture contends with challenges such as limited resources, land fragmentation, and high production costs [2]. These issues have led to~~impacted the competitiveness~~ a comparative disadvantage ~~of~~ in Chinese agricultural exports~~, revealing a comparative disadvantage~~ [3,4] ~~that~~ and ~~hinders~~ hindered export growth and ~~the~~ sustainable development [5].

A sentence in the fifth paragraph of the Introduction section was rephrased as follows:

The main objective of this study is to investigate the evolution, performance, and influencing factors of the export competitiveness network of Chinese provincial agricultural products ~~through the perspective of complex networks~~.

Some sentences in the sixth paragraph of the Introduction section have been rephrased as follows:

This paper, however, examines the international competitiveness of Chinese agricultural products at the provincial level, highlighting~~providing insights into~~ the ~~regionalization and~~ differentiation ~~characteristics of~~ competitive agricultural products across Chinese provinces.

This approach more effectively identifies competitive agricultural products ~~with competitive advantages~~ across~~in each~~ provinces and further enriches the research paradigm ~~and methodology~~ concerning~~regarding the~~ trade relationships.

Using the Exponential Random Graph Model (ERGM), this paper investigates the factors influencing the development of the export competitiveness network ~~of provincial agricultural products~~ from a comprehensive perspective. Compared to the conventional~~traditional linear~~ regression models, ERGM is better suited to track the endogenous dependencies in forming the export competitiveness network~~, a task that conventional regression model models cannot effectively accomplish~~.

A sentence in the fourth paragraph of the Literature review section was rephrased as follows:

Furthermore, rising production costs ~~in China~~ and a diminishing demographic dividend, which ~~the~~ China ~~country~~ once benefited from, ~~are undermining~~ undermine its competitive edge in exporting agricultural products.

A sentence in the second paragraph of the External covariate effect section was rephrased as follows:

By enhancing knowledge of foreign markets [51], international sister-city relationships assist ~~not only in grasping trade and investment opportunities but also~~ in reinforcing identity and trust.

Some sentences in the second paragraph of the Research design section have been rephrased as follows:

This approach adopts topological indicators like loop numbers, node strength, clustering coefficient, and betweenness centrality ~~expands upon social network analysis by~~ to highlight~~ing~~ node heterogeneity, structural complexity, and dynamic variability ~~in network patterns through topological indicators like loop numbers, node strength, average clustering coefficient, and betweenness centrality~~.

Therefore, employing complex network analysis can effectively illuminate the structural evolution of the export competitiveness network ~~of Chinese provincial agricultural products~~ and identify the competitive agricultural products.

In contrast to traditional research methods, complex network analysis can investigate the key factors influencing agricultural export competitiveness at various levels. Based on this, we can provide~~, ultimately providing~~ actionable recommendations to enhance the export competitiveness of these products in Chinese provinces.

A sentence in the fifth paragraph of the Network construction section was rephrased as follows:

Instead, it focuses on the share of specific agricultural exports in the province and the share of national imports of specific agricultural products in total imports.~~competitiveness of a particular agricultural product exported by the province in comparison to all agricultural product exported by that province and those of all other provinces.~~

The paragraph about Node attribute indicators in Empirical analysis was rephrased as follows:

Node attribute indicators. At the provincial level, this paper accounts for GDP, the total output value of agriculture, forestry, animal husbandry, and fishery (AFAHF), and exports [71]. Each province's ~~The~~ GDP and export data for each province are sourced from the China Statistical Yearbook, while the AFAHF data come from the China Rural Statistical Yearbook. Nationally, it is important to account for GDP, population [72], and economic freedom [73] attributes. Each country's~~The~~ GDP and population data ~~for each country~~ are derived from the World Bank’s World Development Indicators database, while ~~data about economic freedom is provided by~~ the Global Heritage Foundation provides data about economic freedom.

A sentence in the third paragraph of the Analysis of regression results section was rephrased as follows:

China has emphasized the importance of promoting high-quality agricultural development, ~~paying more attention to~~ expanding favorable agricultural products ~~in international markets~~, and creating new competitiveness~~competitive advantages~~ ~~in~~ for ~~exporting specialty~~ agricultural products in international markets.

Some sentences in the second paragraph of the Policy implications section have been rephrased as follows:

Enhancing and developing agricultural products with traditional advantages ~~for~~ in Chinese provinces while strengthening collaboration with research institutes and relevant enterprises~~,~~ . ~~which will cultivate distinctive and high-quality varieties and increase the international competitiveness of agricultural products.~~

For example, Shandong Province~~, recognized as a significant agricultural export hub in China,~~ can emphasize its strengths in exporting vegetables and fruits~~. Meanwhile~~, Zhejiang Province can strengthen the international competitiveness of its tea industry, and Yunnan Province can focus on building its strengths in flower exports, among other initiatives.

Some sentences in the last two paragraphs of the Policy implications section have been rephrased as follows:

It is essential to leverage ~~international~~ sister-city relationships to create new opportunities for bilateral economic and trade cooperation and to enhance the competitiveness~~competitive advantage~~ of provincial agricultural exports ~~in friendly countries~~. International agricultural cooperation agreements ~~Interactions and collaborations~~ between provinces and global partners should be encouraged to increase awareness ~~and recognition~~ of provincial agricultural products through bilateral cultural and economic exchanges.

Different regions should carry out reasonable regional planning for agricultural products to avoid competition of homogenized agricultural products in the same market~~homogenization and the proliferation of products that lack competitive strength~~. The eastern region should fully utilize its geographic advantages by targeting~~to consolidate its dominant position in agricultural markets such as~~ the United States, the European Union, Japan, ~~and~~ South Korea, and other countries as the main export destinations for its advantageous agricultural products. Meanwhile, the central and western regions should actively promote the diversification of agricultural export markets based on the China Railway Express and the new western land-sea corridor. By differentiating the layout of agricultural products in different markets, it is easier to form a competitive advantage of agricultural products.

2.Clarity of H3 and H4: The hypotheses related to international sister-city networks and geographic distance (H3 and H4) are interesting, but these sections would benefit from additional theoretical background. Why are sister-city networks expected to play such a crucial role in agricultural product competitiveness? Adding a few supporting references would clarify the logic behind this.

We thank the reviewer for pointing to this important theoretical aspect of our study. In External covariate effect section, we have added some references to support H3 and H4. In the first paragraph:

Individuals can leverage social capital relationships to enhance their advantages and seize opportunities [49].

In the second paragraph:

Sister-city relationships are an advantageous resource [50].

The quality of information and resources transmitted by such strong relationships generated by identification and trust tends to be higher, providing both parties with a competitive advantage in trade and economic activities [43].

Furthermore, it reduces trade disputes and friction, thereby influencing the establishment and robustness of trade relationships between cities and countries [52]. As a result, products from friendly cities or provinces have a significant advantage in entering the markets of friendly countries.

In the third paragraph:

Geographic location influences the development of relationships [22]. Usually, distances are used to proxy costs at an abstract level [53]. As a result, coastal cities possess a greater geographical advantage when exporting their products [54]. Longer ~~However,~~ bilateral geographical distances increase~~elevates the~~ trade costs, thereby becoming an impediment to product exports [55] and further hindering ~~diminishing~~ the establishment of competitively advantageous trade relationships.

3.While the Exponential Random Graph Model (ERGM) is appropriate for the study, the rationale behind choosing this model over other network models should be further elaborated. Highlighting its advantages in capturing interdependencies would reinforce its appropriateness.

The reasons for using ERGM instead of other models have been elaborated upon in the Introduction and Construction of ERGM and variable explanation sections. As proposed by the reviewer, we provide additional explanations.

In the third and fourth paragraphs of Construction of ERGM and variable explanation section, we have made some additions.

It is indeed true that established product export relationships will further accumulate experience for the product's overseas expansion and facilitate its export to additional countries. Newly established export relationships are somewhat dependent on existing ones.

Relative to other network models, such as the Quadratic Assignment Procedure (QAP), which focuses on the correlation and regression between matrices [65], this model does not take into account for the effects of node attributes and covariate networks on relationship development. Similarly, the Simulation Investigation for Empirical Network Analysis (SIENA) primarily incorporates explanatory variables related to structural effects and individual actor attribute effects [66]. However, it also fails to consider external covariate networks.

4. The "star effects" and "node attribute effects" should be linked more explicitly to relevant studies in the literature review. This will ensure that the theoretical foundation for the hypotheses is clear.

As suggested, in the Research on the network of agricultural product export section, we have incorporated new literature related to star effects and node attribute effects.

Countries with high betweenness centrality serve as hubs in agricultural trade networks and tend to establish a greater number of trade relationships [20]. This phenomenon results in more network star effects [21]. Furthermore, the star effect promotes the establishment of additional network relationships [22]. In addition, attribute variables such as factor endowment, economic size, and geographic location are critical factors influencing the expansion of agricultural trade networks [23].

5.While the policy implications section provides useful recommendations, it would benefit from being more specific. For example, how can regional governments leverage these findings to enhance their agricultural competitiveness? Additionally, consider linking the discussion to international trade policies or agreements.

We thank the reviewer for raising this important point. In the second paragraph of Policy implications section, a sentence has been rephrased as follows:

Specifically, provincial governments can improve ~~the quality of~~ agricultural products through standardized, large-scale, and modernized production models, thereby further strengthening the advantages of traditional agricultural products.

We have made some additions in the second paragraph:

Specifically, the government can increase funding and concentrate on fostering scientific and technological innovation within agribusinesses. It is essential to establish a high-quality team of experts in the field of agriculture, consisting of faculty and students from universities, as well as agricultural scientists. Furthermore, emphasis should be placed on fostering collaboration among local governments, agricultural universities, and agriculture-related institutions and enterprises.

Some sentences have been rephrased as follows in the fourth paragraph of Policy implications section:

On the other hand, local governments should leverage major agribusinesses as the foundation for agricultural industrialization to achieve economies of scale.~~provincial governments can enhance the export of advantageous agricultural products by building stable and mature export routes.~~
